# Withdrawal: [Miura A, Otani K, Miyai H, Fukushima H, Matsuishi K. Post‐acute COVID‐19 syndrome with severe sexually deviant behavior. Neuropsychopharmacol Rep. 2022]

**DOI:** 10.1002/npr2.12343

**Published:** 2023-05-07

Withdrawal: [Miura A, Otani K, Miyai H, Fukushima H, Matsuishi K. Post‐acute COVID‐19 syndrome with severe sexually deviant behavior. Neuropsychopharmacol Rep. 2022]

The above article, published online on 07 May 2023 in Wiley Online Library (wileyonlinelibrary.com) as Early View (https://onlinelibrary.wiley.com/doi/10.1002/npr2.12343 ), has been withdrawn by agreement between the authors, the journal Editor in Chief, Tsuyoshi Miyakawa and John Wiley & Sons Australia, Ltd. The Withdrawal has been agreed because the patient revoked consent to the publication.

